# Investigation of the Effect of Inhomogeneous Material on the Fracture Mechanisms of Bamboo by Finite Element Method

**DOI:** 10.3390/ma13215039

**Published:** 2020-11-09

**Authors:** Raviduth Ramful, Atsushi Sakuma

**Affiliations:** 1 Graduate School of Science and Technology, Kyoto Institute of Technology (KIT), Matsugasaki, Sakyo-ku, Kyoto 606-8585, Japan; 2 Mechanical and Production Engineering Department, Faculty of Engineering, University of Mauritius, Reduit 80837, Mauritius; 3 Department of Advanced Fibro-Science, Faculty of Fiber Science and Engineering, Kyoto Institute of Technology (KIT), Matsugasaki, Sakyo-ku, Kyoto 606-8585, Japan; sakuma@kit.ac.jp

**Keywords:** bamboo, fracture mechanisms, inhomogeneous, transversely isotropic, external loading, FEM (finite element method)

## Abstract

Bamboo is a remarkably strong and sustainable material available for construction. It exhibits optimized mechanical characteristics based on a hollow-inhomogeneous structure which also affects its fracture behavior. In this study, the aim is to investigate the effect of material composition and geometrical attributes on the fracture mechanisms of bamboo in various modes of loading by the finite element method. In the first part of the investigation, the optimized transverse isotropy of bamboo to resist transverse deformation was numerically determined to represent its noticeable orthotropic characteristics which prevail in the axial direction. In the second part of this study, a numerical investigation of fracture mechanisms in four fundamental modes of loading, namely bending, compression, torsion, and shear, were conducted by considering the failure criterion of maximum principal strain. A crack initiation stage was simulated and compared by implementing an element erosion technique. Results showed that the characteristics of bamboo’s crack initiation differed greatly from solid geometry and homogeneous material-type models. Splitting patterns, which were discerned in bending and shear modes, differed in terms of location and occurred in the outside-center position and inside-lowermost position of the culm, respectively. The results of this study can be useful in order to achieve optimized strength in bamboo-inspired bionic designs.

## 1. Introduction

**Figure 1:**
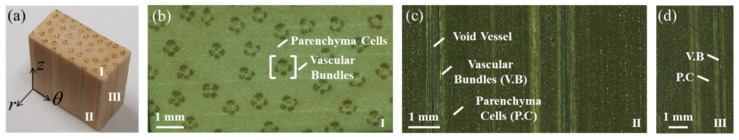
(**a**) Cuboid representation of middle-section, across wall thickness of bamboo culm; (**b**) radial-tangential optical image of bamboo cross-section; (**c**) longitudinal-tangential laser-optical image and (**d**) longitudinal-radial laser-optical image of bamboo longitudinal section. Figure 1b–d were taken by a Keyence VK-X200 series Laser Microscope (Keyence, Osaka, Japan).

Bamboo has been utilized as a prominent construction material in buildings for centuries. Recent trends in using sustainable material for building development is reigniting the interest in natural construction materials. Bamboo can be used as a reliable and sustainable alternative to conventional materials in construction based on two key attributes—namely, a high strength-to-weight ratio, and an unrivalled growth rate of up to 100 cm per day [1,2,3]. Bamboo morphology has developed into a of smart hollow structure consisting of nodes and internodes which provide the structural framework to support the weight of its uppermost section. As a natural composite, it inherits a high strength-to-weight ratio from a hierarchically arranged microstructure composed of concentrated fibers known as a vascular bundle. The axially strong fibers consist of cellulose microfibrils held in a parenchyma matrix composed of hemicellulose and lignin, as shown in Figure 1. Furthermore, the volume fraction of vascular bundles increases with height to compensate for the inferior strength of the uppermost section due to the reduced wall thickness. This inhomogeneous structure enables bamboo to withstand extreme flexural loading caused by wind and snow [1,4,5].

As a natural material, the strength and durability of bamboo depends on several factors such as species, maturity, treatment and loading conditions [6]. In construction, the influence of external loading conditions on its strength has high structural implications. Due to its inhomogeneous material characteristics, bamboo displays a complex fracture behavior. Numerous experimental investigations, conducted on deformation behavior due to bending load, have shown mixed failure modes [7,8,9,10,11]. The failure modes differed in terms of crack initiation and propagation in comparison to other external loading conditions such as compression, shear and torsion conditions as reported in other studies [10,12,13,14,15].

From a fractography analysis, the low interfacial strength reported in bamboo was found to enhance its transverse toughness [16]. The weakened interfacial strength in bamboo, which accounts for its high fracture toughness, is offset by the node and by the optimally distributed microstructure in its cross-section [17]. The resistance against crack propagation parallel to grain, which was governed by interfacial strength, was found to be lower in the outer as opposed to the inner layer [18]. Its inner fibers were found to possess higher toughness as they displayed most of the crack bridging [19,20]. Bamboo was able to absorb greater energy during the large bending deformation of culms [18,21,22,23].

The complex shape and inherent material variability in bamboo poses a major challenge to precisely determine and establish the fracture criterion of full-scale bamboo culm by experimental methods. Numerical methods such as the finite element method (FEM) has an advantage in terms of overcoming the material constraints in bamboo and is an excellent tool to probe further into its mechanical behavior [24]. FEM simulations of full-scale bamboo culm were mainly conducted for the assessment of material limitations and their failure modes under various loading conditions and secondly for a reliability and performance assessment of individual members in complex framework structures [12,24,25,26,27,28].

Even though the fracture toughness of the microscopic structure of bamboo has been investigated in the past [18,19,29,30,31], the effect of material inhomogeneity and unique geometrical attributes on its macroscopic fracture mechanisms remain unexplored to this date to the author’s knowledge. Thus, in this study, the aim is to investigate the effect of material composition and geometrical attributes on the fracture mechanisms of bamboo in various modes of loading by FEM. The results of this study are significant and can be applied to a bamboo-inspired bionic design, which has great prospects for improving the strength in advanced composites [32,33]. By biomimicking the naturally optimized structure of bamboo, artificially optimized structure in complex composites, consisting of axially reinforced fibers and similar macrostructural attributes to bamboo, can be achieved.

## 2. Mechanical Modelling of Bamboo

Bamboo exhibits a high longitudinal-to-transverse bending stiffness ratio which improves its ability to resist externally imposed bending loads. Akin to many other biological structures such as tree trunks, avian and mammalian bones, bamboo’s structure is also optimized to the loading conditions it is subjected to by having an adaptive geometry coupled with an optimally distributed material [24]. This enables smooth deformation in bamboo unique geometry in accordance with the stress- or strain-dependent requirements. In the first part of the study, the optimized transverse isotropy developed in bamboo culm to resist bending deformation was determined numerically by FEM.

### 2.1. Physical Modelling

**Figure 2:**
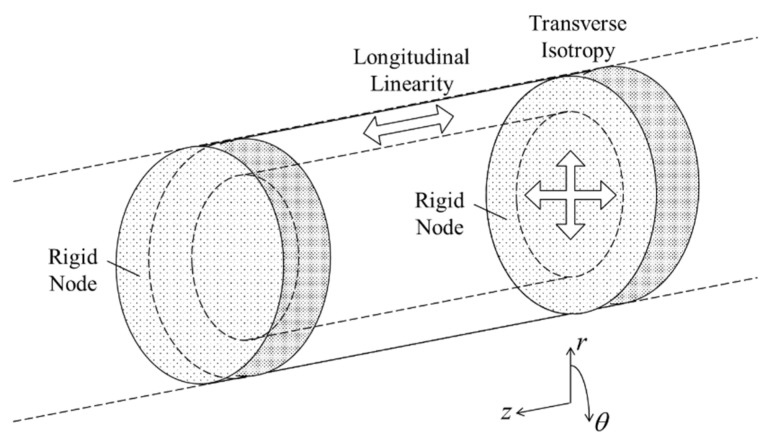
Schematic representation of internodal unit of bamboo.

A representation model of bamboo is needed to analyze its fracture mechanisms since real bamboo has complex characteristics due to species, origin, maturity and moisture content among others. Here, three assumptions for bamboo structure are adopted to analyze the fracture mechanisms with respect to fiber direction. The schematic representation of the assumptions made on the material model, material structure and geometry are shown in Figure 2.

Bamboo behaves non-linearly in conditions of humidity exceeding 60% and linearly in conditions of humidity less than 40% [12]. Non-linear behavior is also widely observed in engineered bamboo [34]. In construction, the linear elastic behavior of bamboo elements is assessed by the seismic response coefficient which accounts for the energy dissipation capacity in bamboo structures [35]. In this study, the linear elastic body was assumed as a constitutive model to clarify the fracture behavior of bamboo [35]. Secondly, the axially strengthened bamboo fibers composed of cellulose microfibrils held in a matrix of hemicellulose and lignin was simplified by assuming a transversely isotropic model [36].

The microstructure of bamboo nodes consists of thickened vascular bundles which are arranged in an interweaving pattern [17]. The nodes in bamboo structure have significant structural importance as they improve the lateral stability and stiffness of slender sections and provide additional support to prevent failure by local buckling in the bamboo culm. Therefore, in this study, the node section, which also represents the undeformed remaining upper section of the bamboo structure, was considered as the strongest part in the bamboo culm and hence modelled as a solid rigid section [7,37,38]. The fracture in the bamboo structure was analyzed and discussed by considering unit-internode modelling to reduce the computational time [37].

### 2.2. Constitutive Modelling

#### Transverse Isotropic Formulation

**Figure 3:**
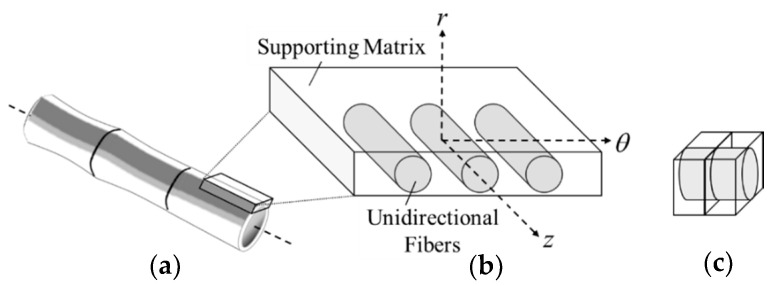
(**a**) Axially strengthened bamboo; (**b**) schematic representation of bamboo lamina and (**c**) transverse isotropy model.

As per second assumption, a transversely isotropic model was considered to replicate the natural composite structure of bamboo composed of axially strengthened fibers, as shown in Figure 3 [39]. In this study, the transversely isotropic model was simply referred to as inhomogeneous model. The internode of bamboo has also been described as a unidirectional long fiber-reinforced composite (Figure 1) since unlike wood, it has no transverse ray cells [17]. Despite its orthotropic nature being well-acknowledged, information about its radial strength is sparsely documented in the literature. Even so, numerous studies have established a link between the tensile, compressive and flexural properties of bamboo and its graded distribution of vascular bundles transversely [21,40,41].

However, as this study mainly focuses on the effects of axially reinforced structure on fracture mechanisms of bamboo, a simplified isotropic material rather than a hierarchically graded material in the transverse direction was considered. Thus, similar to previous studies [12,38], the difference in material constants in the radial and tangential directions of the transversely isotropic model were assumed to be small when compared to the material constants in the axial direction and as a result were considered equal.

For the given lamina structure of anisotropic material, whereby the mechanical properties within one plane is equal in all directions, the three-dimensional representation of stress (*σ*) to strains (*ε*) based on generalized Hooke’s law in contracted notation is written as follows [42]:

(1) $$\sigma_{i}=\;C_{ij}\;\varepsilon_{j},\;i,j=1,\ldots ,\;6$$

(2) $$\varepsilon_{\text{i}}=\;S_{ij}\;\sigma_{j},\;\;\;\;\;\;\;i,j=1,\ldots ,\;6$$

where *C*_ij_ and *S*_ij_ represent the stiffness and compliance matrices, respectively.

Equation (2) is considered since the engineering constants of the compliance matrix (*S*_ij_) can be directly determined in comparison to the ones of the stiffness matrix (*C*_ij_). The transversely isotropic material, which is a special-case orthotropic material, has only five independent engineering constants as represented by the compliance matrix (*S*_ij_) in the following stress-strain relations:

(3) $$\left[\begin{array}{c} \varepsilon_{1}\\ \varepsilon_{2}\\ \varepsilon_{3}\\ \gamma_{23}\\ \gamma_{31}\\ \gamma_{12} \end{array}\right]=\left[\begin{array}{cccccc} S_{11} & S_{12} & S_{13} & 0 & 0 & 0\\ S_{12} & S_{11} & S_{13} & 0 & 0 & 0\\ S_{13} & S_{13} & S_{33} & 0 & 0 & 0\\ 0 & 0 & 0 & S_{44} & 0 & 0\\ 0 & 0 & 0 & 0 & S_{44} & 0\\ 0 & 0 & 0 & 0 & 0 & 2(S_{11}-S_{12})/2 \end{array}\right]\left[\begin{array}{c} \sigma_{1}\\ \sigma_{2}\\ \sigma_{3}\\ \tau_{23}\\ \tau_{31}\\ \tau_{12} \end{array}\right]$$

To represent transverse and longitudinal properties in the isotropic *x*–*y* plane and in the axial direction of *z*-axis, respectively, the engineering constants of compliance matrix (*S*_ij_) are expressed in terms of subscripts T and L as follows:

(4) $$\left[S_{\mathrm{ij}}\right]=\left[\begin{array}{cccccc} \frac{1}{E_{T}} & -\frac{\nu_{T}}{E_{T}} & -\frac{\nu_{L}}{E_{L}} & 0 & 0 & 0\\ -\frac{\nu_{T}}{E_{T}} & \frac{1}{E_{T}} & -\frac{\nu_{L}}{E_{L}} & 0 & 0 & 0\\ -\frac{\nu_{L}}{E_{L}} & -\frac{\nu_{L}}{E_{L}} & \frac{1}{E_{L}} & 0 & 0 & 0\\ 0 & 0 & 0 & \frac{1}{G_{L}} & 0 & 0\\ 0 & 0 & 0 & 0 & \frac{1}{G_{L}} & 0\\ 0 & 0 & 0 & 0 & 0 & \frac{1}{G_{T}} \end{array}\right]$$

where E_L_ and E_T_ are the elastic moduli along the longitudinal and transverse directions, respectively, ν_L_ and ν_T_ are the Poisson’s ratios in the longitudinal and transverse directions, respectively, and G_L_ and G_T_ are the shear moduli in the longitudinal and transverse directions, respectively [42].

### 2.3. Determination of Optimized Transverse Isotropy

In this section, the transverse elastic modulus *E*_T_ was determined numerically on LS-DYNA (Livermore Software Technology, Livermore, CA, USA), an FEM software, by investigating the optimized transverse isotropy developed in bamboo culm to resist bending loads. A numerical simulation was conducted in implicit mode and the maximum principal strain criterion, which is one of the fundamental criteria to evaluate material failure, was adopted to analyze the deformation behavior.

#### 2.3.1. Material Parameters

**Figure 4:**
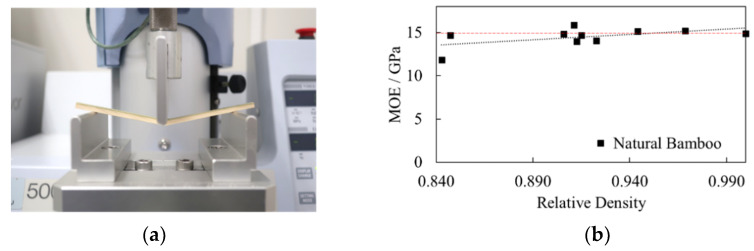
(**a**) Shimadzu EZ-S table-top universal testing instrument used in 3-point bending test; (**b**) 3-point bending test results of natural bamboo.

The first consideration in material constants is the noticeable orthotropic characteristics which prevail in the axial direction of bamboo. The elastic modulus *E*_L_ was experimentally determined by evaluating small clear specimens of Madake bamboo (*Phyllostachys bambusoides*) with average dimensions of 100 mm (longitudinal) × 8 mm (tangential) × 3 mm (radial) in a 3-point bending test on a Shimadzu EZ-S table-top universal testing instrument (Shimadzu Corporation, Kyoto, Japan) as shown in Figure 4a. A total of 10 specimens of natural Madake bamboo were evaluated. The cross-head speed and distance between supports were set at 2 mm/min and 80 mm, respectively, and the supports and punch had radii of 2.5 mm. Tests were conducted in a controlled environment at a temperature of 25 °C and relative humidity below 20%. An average modulus of elasticity (MOE) *E*_L_ of 15 GPa was obtained from experiment, as shown in Figure 4b.

Secondly, bamboo was simplified into a transversely isotropic model. The corresponding longitudinal-to-transverse bending stiffness ratio (*E*_L_-*E*_T_) of this model was initially considered as 100:1 based on past literature data, which reported the longitudinal and transverse stiffness components of bamboo to be less than the mentioned ratio [10,40,43,44]. The material constants in the radial and tangential directions were considered as equal (*E*_T_) which was also the case in previous studies [12,38].

ν_L_ was determined from past literature data as 0.3 while ν_T_ was calculated based on the following orthotropic symmetry condition [25]:

(5) $$\frac{\nu_{\mathrm{ij}}}{E_{i}}=\frac{\nu_{\mathrm{ji}}}{E_{j}},\;i,\;j\;=\;L,\;T\;\left(i\;\neq \;j\right)$$

Additionally, in transversely isotropic material, ν_L_ and ν_T_ must obey the following relations:

(6) $$-1<\nu_{T}<1,-\sqrt{\frac{E_{L}}{E_{T}}}<\nu_{L}<\sqrt{\frac{E_{L}}{E_{T}}},\frac{E_{T}V_{L}^{2}}{E_{L}}<\;\frac{1-\nu_{T}}{2}$$

G_L_ was determined based on the following relation:

(7) $$G_{\mathrm{ij}}=\;\frac{E_{i}E_{j}}{E_{i}+E_{j}+2E_{j}\nu_{\mathrm{ij}}\;},\;i,\;j\;=\;L,\;T\;\left(i\;\neq \;j\right)$$

Since the transverse properties of a transversely isotropic material are not independent, G_T_ was determined from the following expression:

(8) $$G_{T}=\;\frac{E_{T}}{2\left(1+\nu_{T}\right)\;}$$

To compare the deformation behavior of the inhomogeneous structure with other material characteristics, an isotropic material model with elastic properties was considered. In this study, the isotropic model was simply referred to as homogeneous model. The density of both material models was set to 700 kg/m^3^ [45] and their engineering constants are displayed in Table 1.

#### 2.3.2. Geometrical Modelling and Boundary Conditions

The physical model was constructed based on morphological data of Madake bamboo. Hence, for an internode count of 18, an outer diameter, wall thickness and intermodal length of 100, 12 and 450 mm, respectively, were considered [1]. To curtail the computational time, a half-solid cylindrical model was opted to simulate the hollow bamboo structure and, as per the third assumption, the node was assumed as a solid rigid section. The rigid section was assigned with elastic material data.

**Figure 5:**
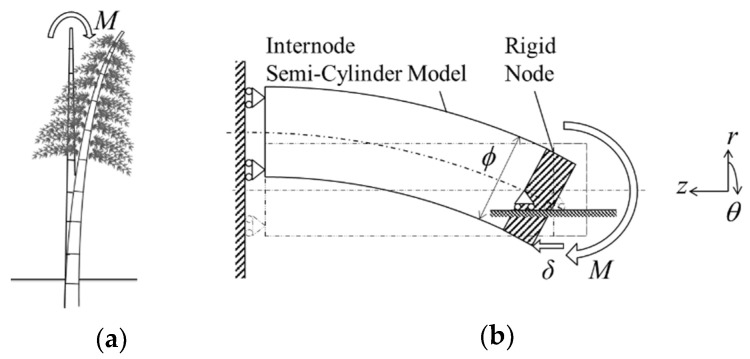
(**a**) Bending deformation due to external loading in natural bamboo; (**b**) boundary condition in bending mode setup.

The optimized transverse isotropy was investigated in bending mode as bamboo is naturally adapted to resist bending loads, as shown in Figure 5a. The boundary conditions of the bending mode are shown in Figure 5b. The bending behavior was simulated by applying a localized displacement δ of 5 mm, aligned with the fiber direction, at the bottom corner of the rigid section, as shown in Figure 5b. A node constraint in the radial direction was applied at the neutral axis of the node-internode intersection. At the wall end, movement was restricted in the z-direction only.

#### 2.3.3. FE Mesh

**Figure 6:**
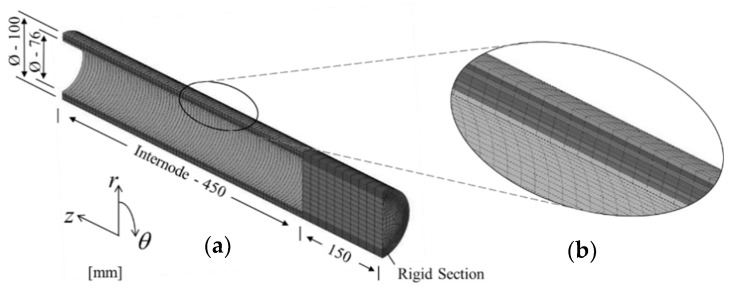
(**a**) Dimension outline of half-solid cylindrical model; (**b**) 8-layer wall-section.

The model was designed on a finite element modelling and postprocessing software (FEMAP) (Siemens Digital Industries Software, Plano, TX, USA), and a hexahedral mesh solid was applied. The meshed domain was discretized into 53,951 nodes and 47,100 elements with finer mesh at the internode section, as displayed in Figure 6a.

#### 2.3.4. Numerical Results and Analysis

**Figure 7:**
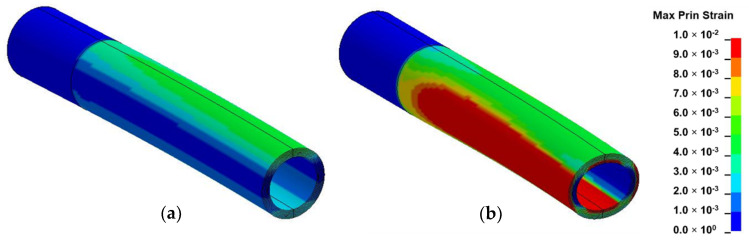
Fringe component of maximum principal strain in: (**a**) homogeneous model; (**b**) inhomogeneous model.

From the numerical results of Figure 7, distinct variation in the circumferential distribution of maximum principal strain was observed between the two material models. As shown in Figure 7a, the original shape of the homogeneous model was found to be preserved as the highest maximum principal strain occurred on its outermost convex section. In contrast to Figure 7b, the highest maximum principal strain distribution in the inhomogeneous model was observed at alternate locations, namely at the neutral axis of the outermost surface and at the inner surface of both convex and concave sides.

Furthermore, the ovalization observed in this material model occurred by inward forces which were generated by longitudinal tensile and compressive strains on convex and concave sides, respectively, during bending. This deformation mode was found to correspond to the natural failure observed in bamboo [7,8,9]. In nature, ovalization is assumed to be more pronounced midway along culm length where the intermodal length reaches a maximum value [1].

**Figure 8:**
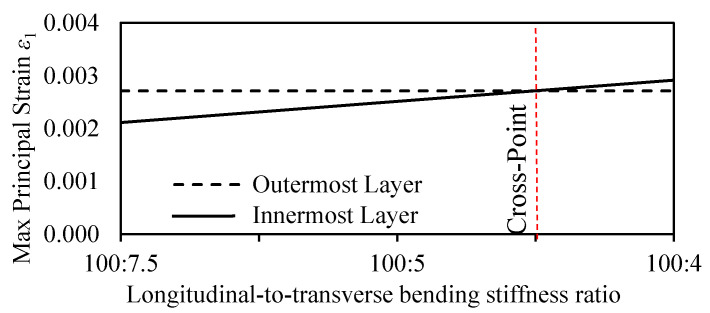
Maximum principal strain *ε*_1_ distribution in inner and outer layers with respect to longitudinal/transverse bending stiffness ratio.

The resistance to bending deformation by the innermost and outermost layers was further analyzed (as shown in Figure 8) to reveal the optimized longitudinal-to-transverse bending stiffness ratio developed in bamboo culm. The maximum principal strain ε_1_ distribution in the inner and outer layers were plotted against a varying longitudinal-to-transverse bending stiffness ratio.

From Figure 8, the analysis of results revealed the occurrence of a simultaneous mixed mode of failure at a longitudinal-to-transverse bending stiffness ratio of 100:4.5. The dotted line at this ratio indicates the cross-point at which both innermost and outermost layers have equal resistance to bending deformation. The equal distribution of deformation resistance between the innermost and outermost layers is assumed to provide bamboo with an optimized structure adapted to withstand high external loadings. Beyond the highlighted point, a shift in maximum deformation is observed from the outermost layers to the innermost layers. The reduction in the transverse strength of the inhomogeneous model accounts for the extra bending toughness observed in bamboo culms during bending.

The optimized longitudinal-to-transverse bending stiffness ratio of 100:4.5 corresponds to actual ratio prevailing between the experimental values of MOE in full-culm bamboo ranging between 20–15 GPa axially and 0.8–0.5 GPa transversely, respectively [10,40,43,44]. As reported by Xu, the transverse tensile strength in orthotropic natural materials ranges between 1/50 and 1/24 of the longitudinal tensile strength due to a significant difference in chemical bond energy as cellulose chain molecules are connected by C–C and C–O axially and by C–H and H–O radially [46]. As a bamboo shoot emerges from the ground, its growth is restricted to the longitudinal direction, as can be observed from its microstructure in Figure 1 due to the absence of cambium [7], hence deriving its strength predominantly in the axial direction. Thus, bamboo has adapted to its natural environment by developing an optimized structure at multiple length scales to withstand external forces [38].

## 3. Investigation of Fracture Mechanisms

Bamboo exhibits a complex deformation behavior when exposed to external loading conditions as a result of its inhomogeneous nature [7,8,9,10,12,13,14,15]. In this section, the bamboo model incorporated with transverse isotropic material was simulated in FEM in external loading conditions, namely bending, compression, shear and torsion modes to investigate its fracture mechanisms.

### 3.1. Fundamental Loading Modes

The simulation of the fundamental loading modes was conducted on LS-DYNA in implicit mode. To investigate crack initiation stage and propagation in each mode of loading, an element erosion technique was implemented. The maximum principal stress and strain criteria were found to be more applicable to assess failure in hard and brittle materials in comparison to ductile ones [47]. Being a hard material, bamboo often exhibits brittle failure which is characterized by sudden split followed by instantaneous crack propagation. The maximum principal strain criterion has previously been used to investigate the failure mechanism in engineered bamboo and timber materials [48], while the maximum principal stress criterion has been considered to investigate the mechanical deformation behavior in bamboo culm sections [12,25]. In this study, the maximum principal strain at failure, ε_max_, was adopted as the failure criterion in the element erosion setting. The ε_max_ criterion, corresponding to fracture initiation by element erosion, was defined as 30% less than the value of the maximum principal strain.

#### 3.1.1. Material Parameters

The corresponding engineering constants of the optimized longitudinal-to-transverse isotropic ratio determined in Section 2.3.4 are displayed in Table 2. The fracture behavior of this inhomogeneous material model was compared with one of homogeneous material characteristics comprising elastic properties, as indicated in Table 1.

#### 3.1.2. Geometrical Modelling and Boundary Conditions

The geometrical models were constructed based on morphological data of Madake bamboo, as in Section 2.3.2, and were effectively refined according to their mode of loading to minimize computational time [1]. A full intermodal length of 450 mm was selected for the bamboo model in compression, torsion and shear modes while a half-length of 225 was selected in the bending mode. A semi-cylindrical model was considered in bending and shear investigations, while full cylindrical and quarter cylindrical models were considered in torsion and compression investigations, respectively. The node was assumed as a full solid with a rigid section 75 mm in length in all simulations except in bending and shear investigations whereby a length of 150 mm was considered.

**Figure 9:**
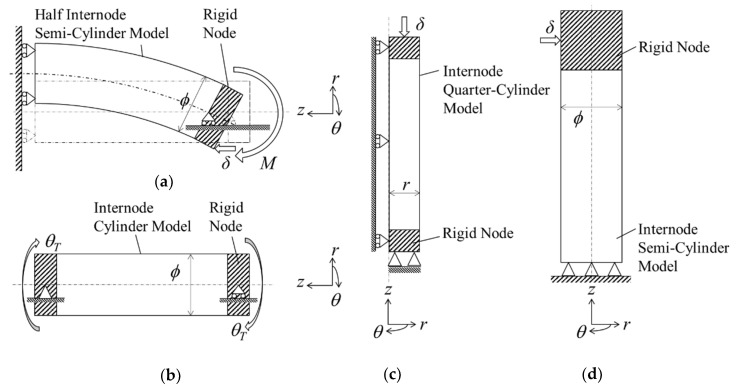
Schematic of boundary conditions of external loading conditions: (**a**) bending mode; (**b**) torsion mode; (**c**) compression mode and (**d**) shear mode.

Specific boundary conditions were applied to each loading mode, as shown in Figure 9. Firstly, constraints perpendicular to the sliced plane were applied to each model to replicate symmetrical sections. Secondly roller supports were applied at the wall end in bending mode while pin supports were applied at the fixed end in compression and shear modes. Thirdly, deformation was simulated by applying a displacement *δ* of 5 mm in bending, compression and shear modes, while an equivalent circumferential displacement in terms of torsion angle θ_T_ of 5.73° was applied in torsion mode.

#### 3.1.3. FE Mesh

For the purpose of comparing FEM results of bamboo-model, four models were considered in each mode of loading and designing on FEMAP. These were (1) solid-homogeneous, (2) solid-inhomogeneous, (3) hollow-homogeneous and (4) hollow-inhomogeneous models. A hexahedral mesh solid was applied throughout all models and a finer mesh of the discretized domain was concentrated at the internode section.

### 3.2. Strain Field Analysis

**Figure 10:**
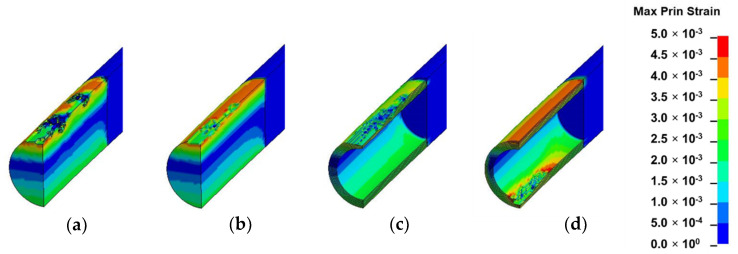
Fringe plots of maximum principal strain of: (**a**) solid-homogeneous; (**b**) solid-inhomogeneous; (**c**) hollow-homogeneous and (**d**) hollow-inhomogeneous models in bending mode.

**Figure 11:**
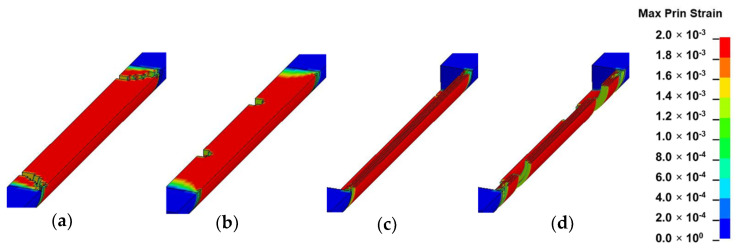
Fringe plots of maximum principal strain of: (**a**) solid-homogeneous; (**b**) solid-inhomogeneous; (**c**) hollow-homogeneous and (**d**) hollow-inhomogeneous models in compression mode.

**Figure 12:**
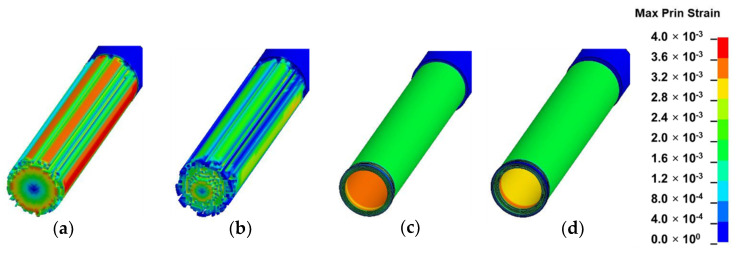
Fringe plots of maximum principal strain of: (**a**) solid-homogeneous; (**b**) solid-inhomogeneous; (**c**) hollow-homogeneous and (**d**) hollow-inhomogeneous models in torsion mode.

**Figure 13:**
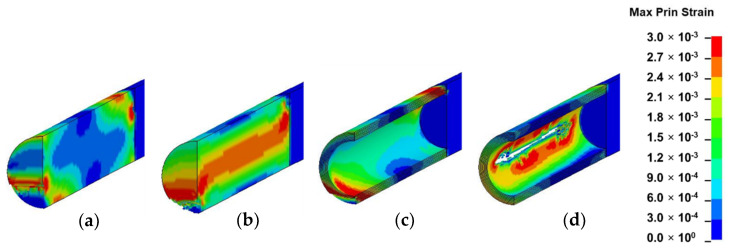
Fringe plots of maximum principal strain of: (**a**) solid-homogeneous; (**b**) solid-inhomogeneous; (**c**) hollow-homogeneous and (**d**) hollow-inhomogeneous models in shear mode.

In this section, the results of each loading mode are presented and discussed based on Figure 10, Figure 11, Figure 12 and Figure 13. The location of crack initiation and the mode of propagation differed significantly as a result of variations in geometry, material property and mode of loading. In solid models, the location of crack initiation in bending, torsion and shear modes of loading were to a large extent similar regardless of material variation. For instance, both solid-type models in shear mode (Figure 13a,b) exhibited break point crack initiation replicating a brittle fracture typically observed in hard solid materials. A notable difference in the compression loading mode of the solid-inhomogeneous model (Figure 11b) was observed as cracks were found to initiate closer to the internode midsection.

The following key observations were made from each mode of loading of the hollow-inhomogeneous model. In bending mode, cracks, which initiated from the lower innermost wall section of the culm, propagated in a longitudinal and radially outward direction in a splitting-like pattern. In the compression mode, cracks originated simultaneously from the outermost wall of the node-internode sections. They followed a diagonal pathway towards the middle of the internode and moved radially inwards. In the torsion mode, however, crack initiation was only observed at the outermost wall section of the prescribed end. Subsequent propagation affected the outermost wall section followed by the node-internode section of the fixed end. Radial penetration was observed at both ends. In the shear mode, a crack was observed to be initiated at the innermost wall of the internode middle section. The crack propagated radially outwards in a splitting-like pattern.

### 3.3. Analysis of Bamboo Fracture Mechanisms

#### 3.3.1. Bending

**Figure 14:**
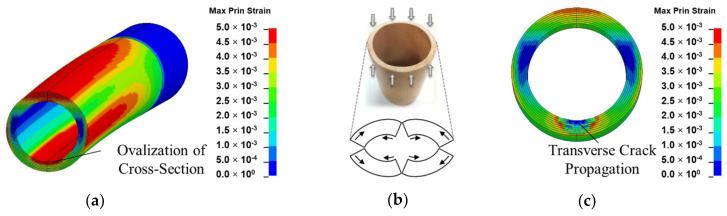
Failure mechanisms of bamboo in bending: (**a**) ovalization of internode cross-section as observed by FEM in bending mode; (**b**) transverse crack propagation originating from 4 vertices in cross-section due to compressive force induced during bending; (**c**) transverse crack propagation in cross-section as observed by FEM in bending mode.

In bending, the crack initiation at the bottom inner wall section of the inhomogeneous culm model is assumed to be related to cross-sectional flattening as a result of poor transverse strength. Cross-sectional flattening corresponds to the deformation theory of tubular section during bending by Brazier’s effect. It is caused by inward forces which are induced by longitudinal tensile and compressive stresses on the convex and concave sections, respectively, and have a tendency to ovalize the cross-section [7,8,9,49]. As deformation is accentuated, ovalization leads to cracking at four vertices, as shown in Figure 14b [44,50]. Splitting also arises in the compressive region of the culm section as fibers are crushed during bending [10].

#### 3.3.2. Shear

**Figure 15:**
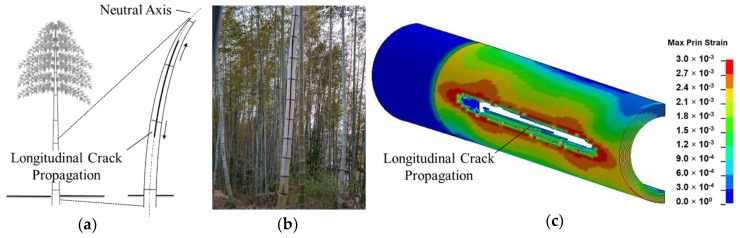
Failure mechanisms of bamboo in shear: (**a**) schematic representation of longitudinal split at culm neutral axis; (**b**) longitudinal split in natural bamboo; (**c**) fringe plot of maximum principal strain of hollow-inhomogeneous model in shear mode.

Shear failure, which was found to occur along distinct shear planes between fibers around the neutral axis (Figure 15a–c), correspond to similar failure mechanisms observed in four-point bending of bamboo culms [10]. The transverse strength in bamboo is further compromised by the inferior interlaminar strength prevailing between fiber bundles within the culm structure [18,29,51]. Bamboo shows excellent ability to resist buckling due to the transverse load during bending and shear despite its radial and tangential stiffness being only about 1/20 of its longitudinal stiffness. The longitudinal fibers of superior strength are conglutinated together by a non-cellulose component. Both MOE results obtained by the experiment and bending and shear simulation results obtained by FEM showed that bamboo structures have a good capacity to resist transverse fractures, given their optimized strength, which stems from their specific organization structures. This uniquely optimized structure enables bamboo to adapt to bending loads caused by snow or wind while at the same time possessing a structure with a high strength-to-weight ratio [17].

#### 3.3.3. Compression

In compression, the crack which initiated from the outermost wall of the node-internode section was found to propagate at an angle of 45°, a mode of failure associated with shear band formation [13]. According to Gauss et al., larger axial strain, which is observed near the middle of the culm at ultimate stress due to restriction by loading platens, will cause the section to bulge-out, leading to a split [15].

#### 3.3.4. Torsion

The observation made in the torsion mode corresponded to the results by Askarinejad et al., which showed that the maximum principal stress distribution of bamboo in torsion in finite element analysis was greatest on its outermost surface [12]. Furthermore, a wider range in the variation of Von Mises stress was observed in bamboo in torsion mode as compared to the homogeneous isotropic model [24].

### 3.4. Normalized Strain Distribution

In nature, the forces subjected in bending and shear modes are significantly higher in comparison to compression and torsion as its hollow structure, consisting of a large length-to-diameter ratio, undergoes substantial flexural deformation by transverse loading. However, in this study a constant displacement was applied to homogenize the boundary conditions prior to simulation in all modes of loading even though the actual case in nature is dissimilar. Even so, from the non-dimensional results of Table 3, bamboo in the bending mode still underwent the largest deformation followed by torsion and shear. The unique geometrical structure of bamboo is thus able to withstand large deformation in the bending mode given their optimum natural design.

Based on the fracture mechanisms obtained by FEM simulations, and in comparison to the observed modes of failure in nature, the most common load combination to which bamboo is subjected is bending and shear loads. Its hollow and large length-to-diameter ratio structure is susceptible to large bending and shear deformations when subjected to lateral loading arising from wind.

### 3.5. Future Recommendation

Other important aspects of bamboo also not investigated in this study, but which significantly contribute to its anti-bending and anti-shearing capacities under transverse load, are its interlaminar strength, transverse hierarchical graded structure and systematically positioned nodes. Further numerical and experimental research involving the effects of these mentioned aspects is required and can be compared with the results of the simple case of the transversely isotropic model in this study to give better insight on fracture mechanisms in bamboo culm.

## 4. Conclusions

In this study, the fracture mechanisms of bamboo were investigated by FEM by considering the noticeable orthotropic characteristics which prevail in its axial direction. The characteristics of bamboo’s crack initiation were found to greatly differ from solid geometry and homogeneous material-type models. In contrast to other geometry-material models which simply displayed break point crack initiation notably in shear mode, distinctive splitting patterns were discerned in the hollow-inhomogeneous models in bending and shear modes. Furthermore, the location of the splitting varied in bending and shear modes which occurred in the inside-lowermost position of the culm and outside-center position, respectively. From the results of this study, it can be concluded that bamboo has developed an optimized transverse isotropic structure to predominantly resist transverse deformation due to bending and shear modes of loading. The fracture mechanisms observed in these modes corresponded to a large extent to failure observed in natural bamboo and as per the previously reported experimental investigations. This study can be furthered to shed light on the deformation mechanisms in artificially crafted complex composites with optimized structures based on bamboo-inspired bionic designs.

## Figures and Tables

**Table 1:** Material parameters used in finite element method (FEM).

Elastic Material Parameters		Orthotropic Material Parameters				
Elastic Modulus (MPa)	Poisson’s Ratio	Elastic Modulus (MPa)		Poisson’s Ratio		Shear Modulus (MPa)
E	ν	E_L_	E_T_	ν_L_	ν_T_	G_L_
15,000	0.3	15,000	150	0.3	0.003	147

**Table 2:** Estimated transverse isotropy properties of bamboo.

Orthotropic Material Parameters				
Elastic Modulus (MPa)		Poisson’s Ratio		Shear Modulus (MPa)
E_L_	E_T_	ν_L_	ν_T_	G_L_
15,000	675	0.3	0.0135	630

**Table 3:** Normalized strain distribution.

Material-Model		Normalized Maximum Principal Strain, ε_1_^1^			
Material Properties	Geometry	Bending Mode	Compression Mode	Torsion Mode	Shear Mode
Inhomogeneous	Hollow	1.000	0.457	0.667	0.577
	Solid	0.779	0.417	0.657	0.670
Homogeneous	Hollow	0.707	0.621	0.672	0.862
	Solid	0.810	0.543	0.662	0.948

^1^ All data are normalized by the greatest value of maximum principal strain (8.25 × 10^−3^) obtained from the hollow-inhomogeneous model in bending mode.
